# Polyarthrite rhumatoïde: la rémission sans biothérapie? L’expérience du Burkina Faso (Afrique de l’Ouest)

**DOI:** 10.11604/pamj.2024.47.12.37537

**Published:** 2024-01-10

**Authors:** Aboubakar Ouédraogo, Fulgence Kabore, Wendlasida Joëlle Stéphanie Zabsonré/Tiendrebéogo, Victor Nonguierma, Binta Savadogo, Kantiga Aida Eudoxie Abassiri, Yamyellé Enselme Zongo, Richard Damade, Dieu-Donné Ouédraogo

**Affiliations:** 1 Rhumatologie, Centre Hospitalier et Universitaire de Bogodogo, Ouagadougou, Burkina Faso,; 2 Médecine Hospitalière et Médecine Interne, Centre Hospitalier Louis Pasteur, Chartres, France

**Keywords:** Polyarthrite rhumatoïde, antirhumatismaux modificateurs de la maladie, réponse thérapeutique, Burkina Faso, Rheumatoid arthritis, disease-modifying antirheumatic drugs, therapeutic response, Burkina Faso

## Abstract

**Introduction:**

l'objectif de cette étude était d'évaluer la réponse thérapeutique des patients traités par les antirhumatismaux modificateurs de la maladie (ARMM) pour une polyarthrite rhumatoïde (PR).

**Méthodes:**

il s ‘agit d’une étude transversale monocentrique descriptive et analytique menée au Burkina Faso de janvier 2019 à décembre 2021 chez des patients atteints d´une PR répondant aux critères American Rheumatism Association/European League Against Rheumatism. La réponse thérapeutique a été évaluée selon les critères de European League Against Rheumatism, le Clinical Disease Activity Index et the Simplified Disease Activity Index après au moins six mois de traitement.

**Résultats:**

cent trois patients dont 85 femmes ont été inclus. La moyenne d´âge des patients était de 50,14 ans ±15,04 ans. Quatre-vingt-sept patients (84,47%) étaient ACPA positif et 50 patients (48,54%) avaient une atteinte radiologique à l´inclusion. Le DAS28-CRP, le CDAI et le SDAI moyen étaient respectivement de 5,17; 32,16 et 35,48 à l´inclusion contre 2,53; 7,83 et 8,76 après au moins six mois de traitement (p<0,0001). Quatre-vingt-seize patients (93,20%) étaient traités par du méthotrexate. Selon les critères de l´European League Against Rheumatism, 89 patients (86,41%) étaient bons répondeurs, 62 patients (60,19%) étaient en rémission. Une amélioration majeure a été observée chez 53 patients (51,46%) selon l´évaluation par le Clinical Disease Activity Index et the Simplified Disease Activity Index.

**Conclusion:**

malgré l´indisponibilité les biothérapies en Afrique sub-saharienne, la rémission de la PR peut être obtenue par une optimisation du traitement par les ARMM et un tight control.

## Abstract (en)

**Introduction:**

the objective of this study was to evaluate the therapeutic response of patients treated with disease-modifying antirheumatic drugs (DMARDs) for rheumatoid arthritis (RA).

**Methods:**

descriptive and analytical single-center cross-sectional study conducted in Burkina Faso from January 2019 to December 2021 in patients with RA meeting American Rheumatism Association/European League against rheumatism criteria. Therapeutic response was assessed according to the criteria of the European League Against Rheumatism, the clinical disease activity index and the simplified disease activity Index after at least six months of treatment.

**Results:**

one hundred and three patients, including 85 women were included. The average age of the patients was 50.14 years ± 15.04 years. Eighty-seven patients (84.47%) were ACPA positive and 50 patients (48.54%) had radiological damage at inclusion. The mean DAS28-CRP, CDAI and SDAI were 5.17, respectively; 32.16 and 35.48 at inclusion compared to 2.53; 7.83 and 8.76 after at least six months of treatment (p<0.0001). Ninety-six patients (93.20%) were treated with methotrexate. According to the criteria of the European League Against Rheumatism, 89 patients (86.41%) were good responders, 62 patients (60.19%) were in remission. Major improvement was observed in 53 patients (51.46%) as assessed by the Clinical Disease Activity Index and the Simplified Disease Activity Index.

**Conclusion:**

despite the unavailability of biotherapies in sub-Saharan Africa, remission of RA can be obtained by optimizing treatment with DMARDs and “tight control”.

## Introduction

La polyarthrite rhumatoïde (PR) est une maladie auto-immune, responsable de destructions articulaires et de manifestations systémiques. Il s´agit du rhumatisme inflammatoire chronique le plus fréquent [1]. On estime qu´elle touche entre 0,18 et 1,2% de la population en Europe [2]. Cette prévalence varie selon les pays et l´ethnie, mais aussi selon que l´on se trouve en zone urbaine ou rurale [2]. En Afrique subsaharienne, la prévalence de la PR varie entre 0,6 et 0,9% [3,4]. Au Burkina Faso, la PR représente 2,84% des patients vus en rhumatologie [5]. Le traitement de la PR a beaucoup évolué ces dernières décennies tant sur le plan des médicaments, avec notamment l´avènement des médicaments biologiques, que sur le plan des stratégies thérapeutiques avec récemment la stratégie du « treat to target » (traiter selon un objectif) et le « tight control » (contrôle strict). L´objectif du traitement est la rémission de la maladie ou l´obtention d´un niveau minimum d´activité tolérable [6-9]. Le traitement médicamenteux initial de la PR fait appel aux antirhumatismaux modificateurs de la maladie (ARMM) en association ou non aux anti-inflammatoires; en cas d´insuffisance de réponse, les médicaments biologiques peuvent être associés [10,11]. En Afrique subsaharienne, le traitement de la PR repose essentiellement sur les ARMM car les conditions socio-économiques rendent les biothérapies inaccessibles aux populations [12,13]. Au Burkina Faso seuls les ARMM sont disponibles pour la prise en charge de la PR. Kénagnon dans son étude sur le bénéfice de la stratégie de suivi par « tight control » au cours de la polyarthrite rhumatoïde rapporte 84% de patients en rémission ou en faible activité selon le Disease ctivity Score 28 (DAS28) [14]. Il existe peu d´études qui évaluent l´efficacité des ARMM en Afrique subsaharienne [15,16]. Le but de ce travail était d´évaluer la réponse thérapeutique après au moins six mois de traitement par les ARMM des patients atteints d´une PR.

## Méthodes

**Cadre de l´étude:** l´étude s´est déroulée dans le service de rhumatologie du CHU de Bogodogo au Burkina Faso (Afrique de l´Ouest). Le service a une capacité de 33 lits (hospitalisation ordinaire et hospitalisation de jour) et réalise des consultations de rhumatologie générale et une consultation hebdomadaire dédiée aux rhumatismes inflammatoires chroniques et aux autres connectivites.

**Plan de l´étude:** il s´est agi d´une étude transversale monocentrique descriptive et analytique menée de janvier 2019 à décembre 2021 afin d´évaluer la repose thérapeutique des patients ayant une polyarthrite rhumatoïde et traités par les ARMM.

**Population d´étude**

**Critère d´inclusion:** tous les patients nouvellement diagnostiqués ou suivis pour une PR active et qui répondaient aux critères ACR/EULAR 2010 ont été colligés [17].

**Critère de non-inclusion:** les patients pour lesquels nous ne disposions pas d´informations sur les données cliniques et *paracliniques* avant la visite d´inclusion (M0) (DAS28, *Clinical Disease Activity Index (CDAI), Simplified Disease Activity Index (SDAI), Health Assessment Questionnaire (HAQ))* ont été exclus de l´étude. Il en était de même pour les patients qui n´avaient pas au moins six mois de traitement par ARMM et refusaient de participer à l´étude.

### Ressources de données et mesures

**Outils de collecte de données:** un questionnaire structure a été administré aux patients lors de la visite d´inclusion et aussi lors des consultations de suivi par un rhumatologue. Les consultations étaient réalisées par des rhumatologues séniors.

**Collecte des données:** les variables démographiques telles que l´âge, le sexe et lieu de résidence ont été collectées. Les données sur le diagnostic, l´activité de la maladie, la durée d´évolution des symptômes, le nombre d´articulations douloureuses, le nombre d´articulations gonflées, la C Reactive Protein (CRP), ont été collectées lors de la consultation initiale ainsi qu´au moment du suivi. Il en était de même pour la posologie et la durée des traitements utilisés. Les résultats des dosages de facteurs rhumatoïdes, des anticorps anti-CCP2, les lésions radiographiques ont été collectées lors de la consultation initiale. Le suivi des patients était mensuel avec un ajustement du traitement selon l´activité de la maladie, puis secondairement plus espacé en cas amélioration. Le DAS28-CRP, le SDAI et le CDAI de chaque patient ont été renseignés lors de chaque consultation de suivi. Une évaluation de la réponse thérapeutique a été réalisée après au moins six mois de traitement (M≥6), selon les critères EULAR et selon la variation du CDAI [18]; critères EULAR (Bon répondeur = DAS28 à JN ≤3,2; Répondeur modéré: 3,2< DAS28 JN ≤5,1, non-répondeur = DAS28 JN >5,1); variation du CDAI (Amélioration majeure sous traitement si diminution de 22 points; Amélioration modérée si la variation du CDAI est comprise entre 10 et 21 points; Pas d´amélioration si <10 points).

**Taille de l´échantillon:** la version 3 de OpenEpi a été utilisée pour calculer la taille de l'échantillon à l'aide de la formule:


$$n=\frac{\left[DEFF*Np\left(1-p\right)\right]}{\left[d^{2}/Z_{1-\alpha /2}^{2}*\left(N-1\right)+p*\left(1-p\right)\right]}$$

Avec un intervalle de confiance (IC) à 95%, une marge d'erreur de 5% et en prenant la proportion de polyarthrite rhumatoïde dans une étude précédente qui était de 2,84% [5]. La taille minimale de l´échantillon était 42. Durant la période d´étude 133 patients ont été colligées.

**Analyse des données:** les données ont été saisies et analysées à l´aide du logiciel SPSS version 25. Les données collectées à M0 et M≥6 ont été comparées à l'aide du test de Student pour les variables quantitatives et un test du Chi^2^ pour les variables qualitatives (seuil de significativité: p < 0,05).

**Considération éthique:** cette étude a été approuvée par le comité d´éthique institutionnel pour la recherche du CHU de Bogodogo par délibération N°2019-01-010. Le consentement éclairé des différents participants a été obtenu avant la collecte des informations. Les informations collectées étaient anonymes et la collecte s´est déroulée dans le respect du secret médical.

## Résultats

**Caractéristiques sociodémographiques:** cent trente-trois (133) patients ayant une PR ont été colligés. Cent trois (103) patients ont été inclus. Il s´agissait de 85 femmes (82,50%) et 18 hommes (17,50%). L´âge moyen était de 50,14 ans ± 15,04 avec des extrêmes de 15 ans et 86 ans. Quatre-vingt-un patients (78,64%) résidaient en zone urbaine et vingt-deux (21,36%) en zone rurale.

**Caractéristiques cliniques et paracliniques:** vingt-huit patients (27,18%) avaient une hypertension artérielle essentielle et sept (6,80%) un diabète sucré. Le délai moyen entre le début des symptômes et la consultation en rhumatologie des patients était de 40,49 mois ±62,628 avec des extrêmes de [0 et 360 mois]. Soixante-cinq patients (63,10%) avaient une CRP élevée (CRP> 6mg). Quatre-vingt-sept patients (84,47%) avaient des anticorps anti-CCP2. Onze patients (10,67%) avaient des manifestations extra-articulaire dont 8 cas (7,76%) de syndrome sec, 2 cas de nodules cutanés et 1 cas d´atteinte pulmonaire. Cinquante patients (48,54%) avaient des lésions structurales à la radiographie à M0 dont 36 (34,95%) une atteinte érosive et 14 (13,59%) une carpite fusionnante. Le DAS28-CRP moyen était de 5,17 ± 1,10 avec des extrêmes de (3,25 et 8,51) à l´inclusion et 2,53 ± 1,01 (0,99 et 6,05) après au moins six mois de traitement (P< 0,001). Le CDAI moyen à M0 était de 32,16 ± 17,38 (7 et 80). Le CDAI moyen à M ≥ 6 était de 7,83 ± 7,92 (0 et 41). Le SDAI moyen à M0 était de 35,48 ± 19,32 (11,20 et 84,87). Le SDAI moyen à M≥6 était de 8,76 ±8,33 (0,02 et 44,40). Le Tableau 1 montre l´évaluation de l´activité de la maladie selon le DAS28-CRP, le CDAI et le SDAI. Le HAQ moyen à M0 était de 1,14 ±0,53 (0 et 2,9). Le HAQ à M≥6 était de 0,22 ±0,12 (0 et 2,6) (p<0,001).

**Tableau 1:** répartition des patients selon l'activité de la maladie

Activité de la maladie selon le DAS28-CRP n (%)						
	**DAS28 (Moyenne ± écart type)**					
		**Activité élevée**	**Activité modérée**	**Activité faible**	**Rémission**	
M0	5,17 ± 1,10	55 (53,40%)	48 (46,60%)	00	00	
M6	2,53 ± 1,01	3 (2,91 %)	20 (19,42%)	18 (17,48%)	62 (60,19%)	
Valeur P	< 0,001					
	**Activité de la maladie selon le CDAI, n (%)**					
	**CDAI (Moyenne ± écart type)**					
M0	32,16 ± 17,38	75 (72,82%)	26 (25,24%)	2 (1,94%)	00	
M6	7,83 ± 7,92	6 (5,83%)	18 (17,47%)	56 (54,37%)	23 (22,33%)	
Valeur P	< 0,001					
	**Activité de la maladie selon le SDAI, n (%)**					
	**SDAI (Moyenne ± écart type)**					
M0	35,48 ± 9,32	68 (66,02%)	35 (33,98%)	00	00	
M6	8,76 ± 8,33	5 (4,85%)	21 (20,39%)	54 (52,43%)	23 (22,33%)	
Valeur P	< 0,001					

**Le traitement:** une corticothérapie a été initiée chez 95 patients (92,23%) avec une posologie moyenne par jour de 18,38 mg ± 10,28 et des valeurs extrêmes de 5 et 80mg. Après six mois de traitement, 22 patients (21,36%) prenaient toujours des corticoïdes avec une posologie moyenne par jour de 5,41mg ± 2,57 (2 et 12mg). Le méthotrexate a été prescrit chez 96 patients (93,20%) à M0 à la posologie moyenne de 15,62mg ± 4,32 par semaine [10 et 20 mg]. Huit patients (7,77%) ont reçu de l´hydroxychloroquine à la dose moyenne de 400mg par jour. Trois patients (2,91%) ont reçu de la sulfasalazine à la dose de 2g par jour et 1 patient du léflunomide à la dose de 10 mg par jour. Parmi les huit patients sous hydroxychloroquine, 5 ont reçu de l´hydroxychloroquine initialement en monothérapie, et les 3 autres patients six mois après en association à un traitement initial par du méthotrexate devant une réponse thérapeutique insuffisante. Il en est de même pour 2 des 3 patients traités par la sulfasalazine. Cinq patients (4,85%) étaient traités par une association d´ARMM à M≥6. Le Tableau 2 montre la répartition de l´activité de la maladie selon l´association thérapeutique utilisée à M≥6.

**Tableau 2:** répartition de l'activité de la maladie selon l'association thérapeutique utilisée après au moins six mois de traitement

	Activité de la maladie selon le disease assessment score of 28 joints-C-reactive protein					
Activité de la maladie	**MTX**	**HCQ**	**SLZ**	**LEF**	**MTX + HCQ**	**MTX + SLZ**
Élevée	3	0	0	0	0	0
Modérée	14	2	0	1	2	1
Faible	18	0	0	0	0	0
Rémission	56	3	1	0	1	1
Total	91	5	1	1	3	2
	**Activité de la maladie selon le clinical disease activity index**					
Élevée	0	0	0	0	0	0
Modérée	15	2	0	1	2	1
Faible	50	0	0	0	0	0
Rémission	21	3	1	0	1	1
Total	91	5	1	1	3	2
	**Activité de la maladie selon le simplified disease activity index**					
Élevée	5	0	0	0	0	1
Modérée	16	0	0	1	2	0
Faible	49	4	0	0	0	0
Rémission	21	1	1	0	1	1
Total	91	5	1	1	3	2

MTX: Méthotrexate, HCQ: Hydroxychloroquine, SLZ: Sulfasalazine, LEF: Léflunomide

**La réponse thérapeutique:** en utilisant les critères EULAR d´évaluation de la réponse thérapeutique, 89 patients (86,41%) étaient bons répondeurs et 9 patients (8,74%) étaient non-répondeurs. Selon le DAS28-CRP 62 patients (60,19%) étaient en rémission. L´évaluation de la réponse thérapeutique par le CDAI et le SDAI rapportait dans les deux cas 53 patients (51,46%) avec une amélioration majeure. Le Tableau 3 montre la répartition des patients selon la réponse thérapeutique. Soixante-dix-huit des 91 patients (85,71%) sous méthotrexate étaient bons répondeurs selon les critères EULAR et 8 patients étaient non répondeurs. Selon le DAS28-CRP 56 des 91 patients (61,54%) traités par du méthotrexate étaient en rémission et 18 patients (20, 23%) avec une faible activité. Le Tableau 4 montre la réponse thérapeutique selon l´association thérapeutique utilisée à M ≥ 6.

**Tableau 3:** répartition des patients selon la réponse thérapeutique

	Effectif (n)	Proportion (n/N)
**Réponse thérapeutique selon les critères EULAR**		
Disease assessment score of 28 joints-C-reactive protein (DAS28-CRP)		
Bon répondeur	89	86,41
Répondeur modéré	5	4,85
Non répondeur	09	8,74
**Réponse thérapeutique selon le clinical disease activity index**		
Clinical disease activity index		
Amélioration majeure	53	51,46
Amélioration modérée	35	33,98
Pas d’amélioration	15	14,56
**Réponse thérapeutique selon le simplified disease activity index**		
Simplified disease activity index		
Amélioration majeure	53	51,46
Amélioration modérée	37	35,92
Pas d’amélioration	13	12,62

**Tableau 4:** répartition des patients selon la réponse thérapeutique et l'association thérapeutique utilisés après au moins six mois de traitement

	Réponse thérapeutique selon les critères EULAR					
	MTX	HCQ	SLZ	LEF	MTX + HCQ	MTX + SLZ
Disease assessment score of 28 joints-C-reactive protein (DAS28-CRP )						
Bon répondeur	78	5	1	1	3	1
Répondeur modéré	5	0	0	0	0	0
Non répondeur	8	0	0	0	0	1
**Total**	91	5	1	1	3	2
**Réponse thérapeutique selon le clinical disease activity index**						
Amélioration majeure	45	5	1	1	1	0
Amélioration modérée	32	0	0	0	2	1
Pas dàmélioration	14	0	0	0	0	1
Total	91	5	1	1	3	2
**Réponse thérapeutique selon le simplified disease activity index**						
Amélioration majeure	44	5	1	1	1	1
Amélioration modérée	35	0	0	0	2	0
Pas d’amélioration	12	0	0	0	0	1
**Total**	91	5	1	1	3	2

MTX: méthotrexate, HCQ: hydroxychloroquine, SLZ: sulfasalazine, LEF: léflunomide

## Discussion

La rémission de la PR sans biothérapie est un objectif accessible. Selon les critères EULAR et quel que soit le schéma thérapeutique utilisé, 86% des patients de notre étude étaient bons répondeurs aux traitements par ARMM avec 62 patients en rémission et 18 patients avec une faible activité. Ces résultats sont favorables et traduisent une efficacité des ARMM dans notre série, malgré un retard de consultation important (40,49 mois) responsable de formes cliniques évoluées observées chez beaucoup de nos patients. L´association méthotrexate avec de faible dose de corticoïde à l´initiation du traitement fait partie de toutes les recommandations actuelles de la prise en charge de la PR [10,11]. Le méthotrexate a été largement l´ARMM le plus prescrit dans notre étude, chez plus de neuf patients sur 10 (93,20%). Il était associé à une corticothérapie chez 8 patients sur 10 lors de l´initiation du traitement, permettant d´obtenir une bonne réponse thérapeutique chez plus de 8 patietns sur dix, selon les critères EULAR. En Afrique sub-saharienne, le méthotrexate est l´ARMM le plus utilisé dans la prise en charge de la PR comme rapporté par Ouédraogo *et al*. au Burkina, Ndongo *et al*. au Sénégal et Hodkinson *et al*. en Afrique du sud avec respectivement 52,38%; 66,83% et 63,4% de patients traités [5,15,16]. Le méthotrexate, efficace et peu coûteux reste le gold standard dans le traitement de la polyarthrite rhumatoïde [19]. Les associations thérapeutiques d´ARMM ont été peu utilisées dans notre étude avec seulement cinq patients dans ce schéma thérapeutique. L´association méthotrexate et hydroxychloroquine était la plus utilisée, chez 3 patients, permettant d´obtenir la rémission pour 1 patient. La bonne réponse thérapeutique chez la plupart des patients avec la monothérapie d´une part, mais aussi la faible disponibilité de certains ARMM d´autre part peuvent expliquer ce faible nombre de patients traités par les associations d´ARMM. Les associations thérapeutiques d´ARMM utilisées d´emblée comme traitement initial n´ont pas fait la preuve d´une efficacité supérieure au méthotrexate en monothérapie, en association ou non aux glucocorticoïdes [19]. Toutefois, les combinaisons progressives d´ARMM en association avec les glucocorticoïdes semblent avoir une efficacité supérieure à la monothérapie [20]. Dans la littérature africaine, quelques études rapportent l´utilisation des associations thérapeutiques avec des résultats divers [15,16]. En effet les méthodes d´évaluations de la réponse thérapeutique ainsi que la posologie et le type d´association d´ARMM utilisée pourraient expliquer cette différence dans les résultats. Le méthotrexate restant la pierre angulaire de la plupart des associations d´ARMM utilisées.

La réponse thérapeutique dans notre étude est largement supérieure à celle retrouvée dans la littérature en Afrique sub-saharienne; en effet, Ndongo *et al*. au Sénégal rapportaient 53,9% de patients bons répondeurs après un suivi de 6 mois selon les critères EULARD [15]. Carter *et al*. ainsi qu´Hodkinson *et al*. en Afrique du Sud rapportaient respectivement 3,1% de rémission selon le CDAI et 10,4% de rémission selon le SDAI contre 22,33% de rémission dans notre série. Au-delà des ARMM utilisés, la stratégie de suivi des patients contribue énormément à l´obtention de meilleures réponses. En effet Kenagnon dans son étude sur la stratégie du « tight control » (contrôle strict) dans la PR rapportait 84% de patients en rémission ou en faible activité [14]. Drosos A *et al*. concluaient à ce propos que la stratégie de traitement était plus importante que les médicaments utilisés dans la polyarthrite rhumatoïde [9]. La stratégie de « contrôle strict » dans le suivi des patients mise en œuvre dans notre étude pourrait expliquer cette différence de résultats avec les autres auteurs. En effet une décennie s´est écoulée entre ces études et la nôtre et les conditions d´accessibilité aux biothérapies n´ont pas foncièrement évolué. Mais la meilleure accessibilité aux ARMM notamment au méthotrexate, le nombre accru de spécialistes en rhumatologie et de façon plus générale l´amélioration de l´offre de soin sont sans doute d´autres facteurs qui ont contribué à l´obtention de nos résultats favorables. Un dixième des patients évalués étaient non répondeurs selon les critères EULAR. Il s´agit de patients nécessitant l´introduction d´un médicament biologique afin d´obtenir un meilleur contrôle de la maladie. Ce chiffre reste important et rappelle la nécessité de rendre accessible les médicaments biologiques qui sont indispensables pour dans la prise en charge de ces patients non-répondeur aux ARMM. Østergaard M *et al*. Rapportent 39,2% de rémission selon le CDAI à 12 mois de traitement par les ARMM contre 59,3% de rémission avec l´Abatacep; 52,3% de rémission avec le Certolizumab et, 51,9% de rémission avec le Tocilizumab, tous prescrits en association avec le méthotrexate [21]. Ces résultats confirment l´efficacité démontrée des médicaments biologiques dans le traitement de la PR, dont le coût reste le principal obstacle à leur utilisation en Afrique sub-saharienne comme dans beaucoup d´autres régions du monde.

## Conclusion

Les résultats du traitement de la PR par les ARMM au Burkina Faso sont favorables, permettant d´atteindre une bonne réponse thérapeutique après au moins six de traitement pour la majorité des patients. Le méthotrexate est l´ARMM le plus utilisé dans notre série. Seule une faible proportion de patients étaient non-répondeurs et avaient besoin d´un renforcement du traitement par des médicaments biologiques. Le « tight control », la réduction du délai diagnostique ainsi que l´utilisation de combinaisons thérapeutiques d´ARMM devraient permettre, dans les pays d´Afrique sub-saharienne, l´obtention de la rémission de la PR ou l´activité la plus faible possible en attendant la disponibilité des biothérapies qui sont incontournable pour au moins un patient sur dix.

### Etat des connaissances sur le sujet

- *Le méthotrexate est le traitement de première intention dans la polyarthrite rhumatoïde;*
- *Les biothérapies sont nécessaires en cas d´insuffisance de réponse des disease-modifying antirheumatic drugs*.

### Contribution de notre étude à la connaissance

- *Les résultats du traitement de la polyarthrite rhumatoïde par les antirhumatismaux modificateurs de la maladie sont meilleurs que ceux obtenus il y´a une décennie malgré que les conditions d´accessibilité aux biothérapies n´aient pas foncièrement évolués;*
- *L´optimisation du traitement par les DMARDs et l´utilisation de la stratégie du « tight contrôle » dans le suivi, est une bonne alternative pour obtenir la rémission de la polyarthrite rhumatoïde en attendant l´arrivée des biothérapies dans notre contexte de sous-développement*.
